# Visual Hallucinations With Arsenic Trioxide Therapy in Acute Promyelocytic Leukemia

**DOI:** 10.7759/cureus.66672

**Published:** 2024-08-12

**Authors:** Himil J Mahadevia, Ammar Al-Obaidi, Furha Cossor

**Affiliations:** 1 Internal Medicine, University of Missouri Kansas City School of Medicine, Kansas City, USA; 2 Hematology/Oncology, University of Missouri Kansas City School of Medicine, Kansas City, USA; 3 Hematology, Saint Luke's Hospital, Kansas City, USA

**Keywords:** all-trans retinoic acid (atra), acute promyelocytic leukemia (apml), neurological toxicity, visual hallucinations, arsenic trioxide

## Abstract

A 68-year-old male with a history of diabetes and hypertension was diagnosed with acute promyelocytic leukemia (APML). He underwent induction therapy with all-trans retinoic acid (ATRA) and arsenic trioxide. He had a complete hematologic response and was initiated on consolidation therapy with arsenic trioxide (0.15 mg/kg/day intravenous (IV)) and ATRA (45 mg/per meter square of body surface area/day IV). He developed blurred vision and floaters after a few days. Soon after, he felt that his diabetic neuropathy had suddenly worsened. The floaters and flashing lights worsened and morphed into visual hallucinations. He reported seeing figures watching him from the corner of the room. He was admitted and head imaging was unremarkable. Routine labs did not show anything unusual. Arsenic trioxide therapy was held. The hallucinations gradually started decreasing and eventually subsided after around eight weeks. ATRA was continued but arsenic was permanently discontinued. Arsenic is known to cause poisoning if exposed in significant amounts. The arsenic dose used for APML is substantially low (0.15 mg/kg/day IV). We delineate this unanticipated case of arsenic toxicity leading to severe neurological symptoms like visual hallucinations which has not been previously reported in the literature. It is imperative to closely monitor patients who are on arsenic therapy and inform them about possible rare toxicities.

## Introduction

Arsenic is a naturally occurring element and poisoning may occur if it is ingested in large amounts. Arsenic toxicity is characterized initially by gastrointestinal symptoms like nausea and watery diarrhea, and rare serious manifestations often include cardiac arrhythmias, hypotension, and bleeding diathesis [1]. Arsenic has neurotoxic properties and can affect the peripheral as well as the central nervous system. Neurological side effects may develop within a few hours after exposure but are often noticed 2-8 weeks after exposure [2]. Peripheral toxicities usually involve symmetrical sensorimotor neuropathy characterized by numbness, pain, and paresthesias, especially in the lower extremities [2]. Central nervous system manifestations include headache, vertigo, somnolence, confusion, delirium, seizures, and coma [3]. Furthermore, psychiatric symptoms like depression, anxiety, and psychosis may also occur [3]. The minimum lethal dose is usually estimated to be 1 mg/kg [4].

Arsenic trioxide is an old drug in modern medicine that has demonstrated excellent clinical activity against acute promyelocytic leukemia (APML) harboring t(15;17) PML-RARA translocation [5,6]. It is currently preferred as a front-line regimen for the induction treatment of low-risk APML along with all-trans retinoic acid (ATRA) [7]. Randomized controlled trials have demonstrated the superiority of the arsenic plus ATRA combination compared to either therapy alone [8-10]. Arsenic at therapeutic doses (standard dose: 0.15 mg/kg/day intravenous (IV)) is generally well tolerated, and serious adverse events are rare [8,9]. We hereby delineate a very unusual case where the patient received arsenic trioxide at a standard dose (0.15 mg/kg/day IV) during induction treatment along with ATRA (45 mg/per meter square of body surface area/day IV) and was then initiated on consolidation therapy (CNT) with both drugs. Several days after commencing the CNT with ATRA plus arsenic trioxide, the patient started having visual symptoms and worsening neuropathy and eventually developed vivid visual hallucinations.

## Case presentation

**Figure 1:**
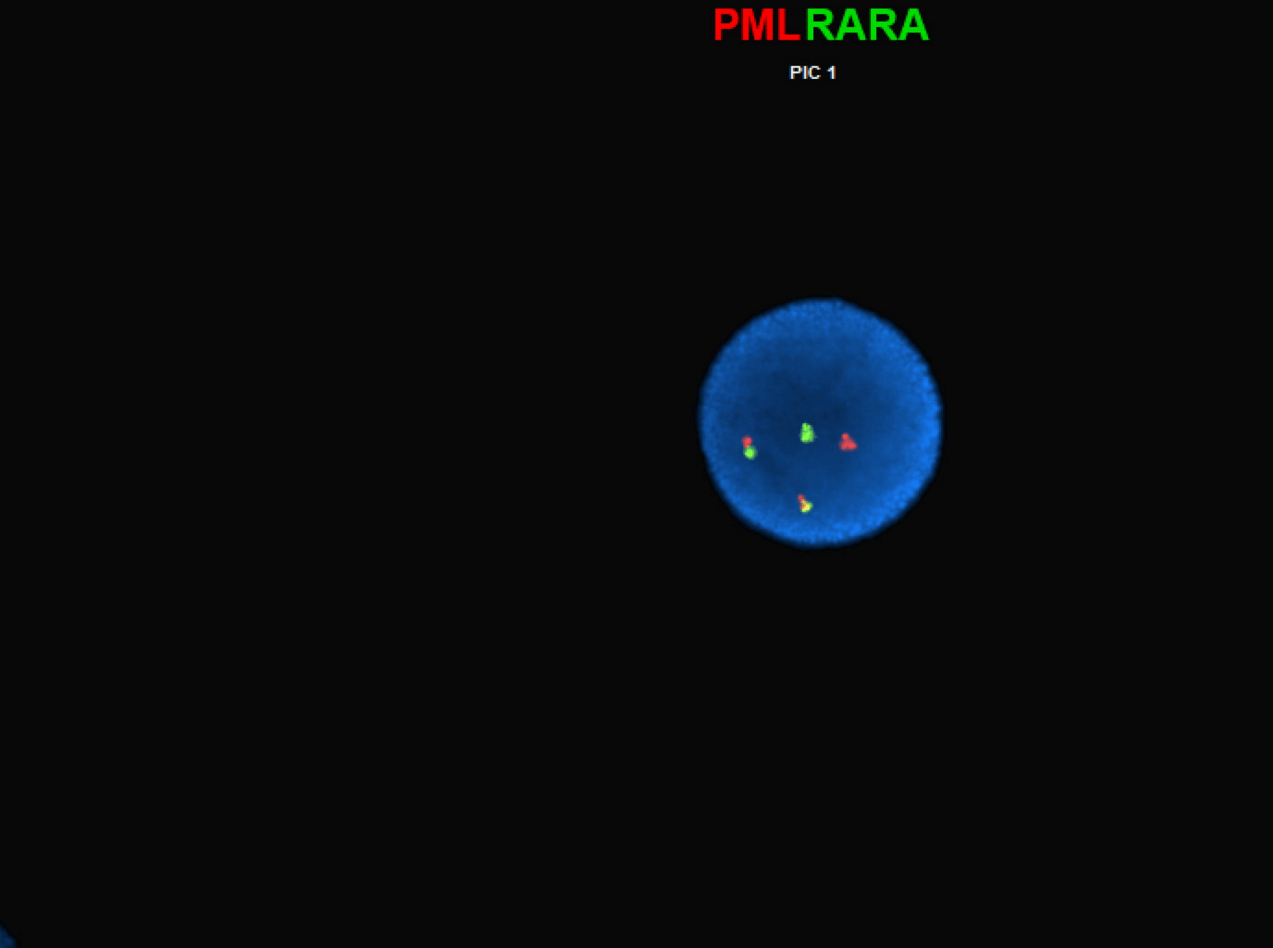
FISH test showing t(15;17) PML-RARA fusion FISH: fluorescence in situ hybridization

A 68-year-old male with a history of diabetes, mild diabetic neuropathy, and hypertension was found to have new-onset pancytopenia on routine annual blood work. The initial investigations were unrevealing. Subsequently, the patient underwent bone marrow biopsy which was consistent with APML. The patient had marked hypercellular marrow with more than 95% promyelocytes and substantially reduced multilineage hematopoiesis. Fluorescence in situ hybridization (FISH) analysis for t(15;17) PML-RARA was positive, and flow cytometry revealed 71% blasts (Figure 1). He was urgently hospitalized for induction therapy. ATRA at a dose of 45 mg/per meter square of body surface area/day IV in divided doses and arsenic trioxide 0.15 mg/kg/day IV were initiated along with 60 mg of prednisone to prevent differentiation syndrome. He developed a 5% weight loss and required a reduction in arsenic trioxide dose. He had a complete hematologic response with bone marrow biopsy on day 28 showing 2% normal-appearing blasts and normal phenotype on flow cytometry.

He was subsequently initiated on CNT with arsenic trioxide 0.15 mg/kg/day IV five days a week for four weeks and ATRA 45 mg/per meter square of body surface area/day IV for two weeks. He developed blurred vision and floaters after a few days. He was seen at an outpatient eye center and was found to have no acute issues except cataracts. After a review of the literature, it was discovered that arsenic trioxide therapy could cause vision changes in up to 10% of cases and was not an indication to discontinue therapy. Soon after, he felt that his diabetic neuropathy had suddenly worsened with accentuated numbness and pain in the heels. There was consideration to hold arsenic trioxide for two weeks to evaluate if that was the cause and initiate gabapentin therapy. Arsenic trioxide therapy was continued per the patient's wishes. His neuropathy continued to deteriorate. The floaters and flashing lights worsened and morphed into visual hallucinations. He reported seeing figures watching him from the corner of the room. He also acknowledged that he felt confused about the layout of places and expressed difficulty in figuring out the way to go home. He was admitted and a head CT scan showed no acute intracranial process. Laboratory workup including complete blood count and complete metabolic profile was unremarkable. A lumbar puncture and CSF analysis were unrevealing. Arsenic trioxide therapy was held. His other medications were reviewed and did not seem to contribute to neurological symptoms. The hallucinations gradually started decreasing and became more intermittent. He was subsequently discharged.

Arsenic trioxide therapy was permanently discontinued and the patient was initiated on gabapentin. In a subsequent outpatient follow-up visit four weeks later, the patient noted a decreasing frequency of hallucinations, and his vision changes had ameliorated. The numbness and pain in the hands and feet improved with gabapentin therapy. The hallucinations eventually resolved, and neuropathy improved significantly in the next follow-up visit after around four weeks. CNT for APML with ATRA alone was continued for a total of seven cycles. The patient remained in complete molecular remission.

## Discussion

Arsenic trioxide is used at a dose of 0.15 mg/kg/day along with ATRA at a dose of 45 mg/m^2^/day in divided doses during induction therapy until bone marrow remission is noted [7]. During CNT, arsenic trioxide at a dose of 0.15 mg/kg/day IV is administered for five days a week for four weeks. This is repeated every eight weeks for a total of four cycles, and ATRA at a dose of 45 mg/m^2^/day is given for two weeks, every four weeks for a total of seven cycles [7]. This therapy lifts the differentiation blockade and allows the maturation of leukemic cells [11]. It also possesses direct apoptotic effects [12]. Wu et al. reported an unusual case of acute psychosis, hallucinations, and obsessive-compulsive symptoms with arsenic poisoning due to chronic occupational exposure who responded well to a combination of antipsychotics plus antidepressants [13]. However, visual hallucinations have not been previously reported in the literature with arsenic trioxide at therapeutic doses used in APML (0.15 mg/kg/day).

Arsenic-induced neurotoxicity is mainly through oxidative stress, increased apoptosis in cerebral neurons as well as increased destabilization, disruption of cytoskeletal framework, and axonal degeneration [3,14]. It can induce changes in the mitochondrial membrane integrity and reduce membrane potential in the neuronal cells [14]. These alterations facilitate unregulated production of superoxide anion subsequently resulting in the formation of free radicals. Arsenic inhibits succinic dehydrogenase activity which also enables the buildup of oxidative stress [14]. Electrophysiological studies performed on patients with arsenic-induced peripheral neuropathy demonstrated a reduced nerve conduction velocity typical of axonal degeneration [2]. The pharmacokinetics of arsenic trioxide is not definitively known. Arsenic trioxide metabolism involves the reduction of pentavalent arsenic to trivalent arsenic by arsenate reductase enzyme [15]. Trivalent arsenic further undergoes methylation by methyltransferase enzymes mainly in the liver [15]. Thus, arsenic trioxide requires dose reduction in patients with hepatic impairment. Arsenic trioxide is a substrate for multidrug resistance-associated protein (MRP) and p-glycoprotein (p-gp). Medications that strongly inhibit these transporters may attenuate the efflux of arsenic and enhance its tissue concentrations. Our patient had no prior history of central nervous system disease. His liver function tests were unremarkable. He was not on medications that exert potent inhibition of MRP/p-gp. He denied using any herbal supplements. He had anticipated minor adverse events with induction therapy which included arsenic trioxide. However, after initiating CNT, the patient developed worsening neuropathy and vivid visual hallucinations. Holding arsenic trioxide therapy gradually led to improvement.

There may be genetic predispositions or environmental factors leading to altered pharmacokinetics or pharmacodynamics of arsenic trioxide that enhance the risk of developing neurological toxicity which are yet to be determined. Also, a study noted that arsenic toxicity is dependent on multiple factors like chemical species, valance state, uptake, and accumulation [16]. It is essential to definitively understand the importance of arsenic metabolism and the biological functions of its metabolites to further characterize toxicity profiles [16]. Although our patient improved with the cessation of arsenic therapy, chelation therapy may be considered if there is a lack of improvement. A case study reported a 62-year-old female with refractory APML who was treated with arsenic trioxide and developed a multi-level cardiac conduction block [17]. She was treated with dimercaprol chelation therapy, and her conduction abnormality resolved [17].

## Conclusions

Arsenic trioxide will continue to remain the mainstay treatment along with ATRA for low-risk APML. It is imperative to provide guidance and education to patients and ancillary staff about potential neurological adverse events before initiating arsenic trioxide therapy for APML. Identifying and managing toxicities related to arsenic trioxide therapy early on together with monitoring blood arsenic concentration whenever toxicity is suspected should be a significant component of APML management to improve overall patient outcomes.
